# Systemic air embolism complicating upper gastrointestinal endoscopy: a case report with post-mortem CT scan findings and review of literature

**DOI:** 10.1080/20961790.2016.1252898

**Published:** 2017-01-16

**Authors:** Zabiullah Ali, Ferdia Bolster, Eric Goldberg, David Fowler, Ling Li

**Affiliations:** a Office of the Chief Medical Examiner, State of Maryland, Baltimore, MD, USA; b Sino-US Forensic Evidence Science Research Center, Collaborative Innovation Center of Judicial Civilization, China University of Political Science and Law, Beijing, China; c Division of Forensic Pathology, University of Maryland School of Medicine, Baltimore, MD, USA; d Department of Radiology, Mater Misericordiae University Hospital, Dublin 7, Ireland; e Division of Gastroenterology and Hepatology, University of Maryland School of Medicine, Baltimore, MD, USA

**Keywords:** Forensic science, forensic pathology, air embolism, endoscopy, digestive system, pancreaticoduodenectomy, post-mortem computed tomography scan

## Abstract

Endoscopy of the gastrointestinal and biliary tract is a common procedure and is routinely performed for therapeutic and diagnostic purposes. Perforation, bleeding and infection are some of the more common reported side effects. Air embolism on the other hand, is a rare complication of gastrointestinal endoscopy. We report a 77-year-old African-American female with a history of pancreatic cancer, which was resected with a Whipple procedure. As part of diagnostic and therapeutic procedure, an endoscopic retrograde cholangiopancreatography was planned several months after the surgery. The patient's heart rate suddenly slowed to 40 bpm during the procedure and she became cyanotic and difficult to oxygenate after the endoscope was introduced and CO_2_ gas was insufflated. A forensic autopsy was performed with post-mortem computed tomography (PMCT) and revealed extensive systemic air embolism. The detailed PMCT and autopsy findings are presented and current literature is reviewed.

## Introduction

Air embolism is a life-threatening medical condition with a high mortality rate. Air embolism is a rare complication of any gastrointestinal (GI) endoscopic procedure and requires a high clinical suspicion to make a diagnosis. Furthermore, the diagnosis must be made rapidly to prevent a potentially fatal outcome.

Air embolism can due to surgical procedures, especially neurosurgical intervention and intravascular procedures, scuba diving, penetrating trauma such as stab wounds or gunshot wounds, blunt trauma, cardiopulmonary resuscitation among others [1–3]. Although rare, air embolism can result from any endoscopic procedure, but is most commonly associated with endoscopic retrograde cholangiopancreatography (ERCP) [4]. Risk factors include previous surgeries of pancreas and biliary system, cholangitis, and inflammatory bowel disease [5].

The mechanism by which air is introduced into the blood circulation is debated, but many investigators believe that mucosal damage due to high pressure air, biliary venous fistula and transection of duodenal vein radicles among others may be the underlying mechanism for the introduction of intravascular air [4,6].

In forensic medicine, detection of air embolism is important for determination of cause and manner of death. There are different methods used to identify the presence of gas in the right chamber of the heart, such as puncturing the right ventricle under the water [7], aspirating the gas with a spirometer and analysing the gases [8], and more recently, utilizing multi-detector computed tomography [9]. Regardless of the methodology used, the presence of putrefactive gas should be considered and differentiated from air embolism.

## Clinical history and course

The decedent was a 77-year-old African-American female with a history of pancreatic cancer, which was resected approximately nine months previously with a Whipple procedure. She subsequently underwent radiation and chemotherapy. She had multiple readmissions for hematemesis and melena, and had several upper GI endoscopies and colonoscopies, which showed “gastropathy” and no active bleeding sites, although a bleeding site had been identified on Tcm99-NM RBC (technetium 99 nuclear medicine labelled red blood cell) scan. During her last admission, she presented with septicemia and was diagnosed with mycotic thrombus of the retrohepatic inferior vena cava (IVC) and portal vein. She underwent an AngioJet thrombectomy with partial removal of the mycotic thrombus, but later relapsed with a re-accumulation of mycotic thrombus at the IVC and she remained septic. Subsequently, an abdominal MRI scan was performed and showed borderline intrahepatic biliary dilatation to the hepaticojejunostomy with mural enhancement and mild, but diffuse bile duct thickening, suggestive of possible cholangitis. An ERCP was to further evaluate planned for the following day.

The endoscopy procedure was complicated due to previous surgical intervention. A TJF – 160VF-443 Olympus® duodenoscope was introduced under general anaesthesia through the mouth and advanced to the afferent limb of the jejunum. Normal air was used instead of CO_2_ for insufflation. There was evidence of a patent gastrojejunostomy consistent with previous pancreaticoduodenectomy. The afferent limb was easily traversed, although the limb was tortuous to the point where a decision was made to not continue to traverse with a duodenoscope. The scope was withdrawn with a goal of exchanging for a paediatric colonoscope. Before insertion of the colonoscope, the patient's heart rate slowed to 40 s and she became dusky and difficult to oxygenate. A bedside echocardiography revealed air in the right ventricle of the heart. A code was immediately started, but the patient could not be resuscitated.

## Post-mortem investigations

**Figure 1.**
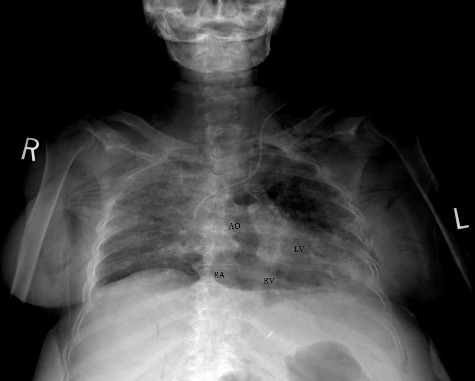
Lodox anterior-posterior view: there is intravascular and intracardiac air in the aorta (AO), right atrium (RA), right ventricle (RV) and left ventricle (LV).

A forensic autopsy was requested. A full body Lodox stat scan was performed prior to the post-mortem computed tomography (PMCT) scan, which was suggestive of air in the right side of the heart, but the results were equivocal due to large patient size and overlying soft tissues (Figure 1). A non-enhanced PMCT was performed 15 hours post-mortem with general electronic (GE) light-speed^16^ 16 row-detector scanner. The head and neck were scanned with a slice thickness of 0.625 mm, tube configuration 140 kV and 480 mA, rotation time 2 s, field of view 25 cm, detector configuration 16 mm × 1.25 mm and beam collimation 10 mm. The chest, abdomen, pelvis and extremities were scanned with a slice thickness of 1.25 mm, tube voltage 140 kV and 364 mA, rotation time 1 s, pitch 0.938:1, detector configuration 16 mm × 1.25 mm, beam collimation 20 mm and maximum field of view 65 cm. All images were reconstructed using the GE advanced work station (AW version 2.5-5) (General Electric, Milwaukee, WI, USA) and an Aquarius workstation (Terarecon, Foster City, CA, USA). The images were interpreted by a radiologist with experience in forensic radiology and a forensic pathologist with five years of experience in forensic radiology and a clinical radiology background.

**Figure 2.**
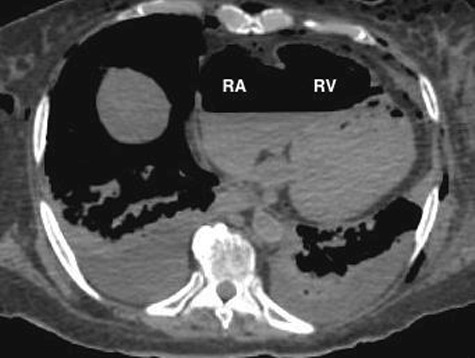
Axial CT of chest demonstrates large amount of air within the right atrium (RA) and right ventricle (RV) (lung window).

**Figure 3.**
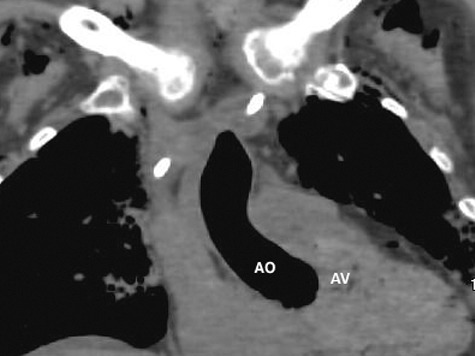
Coronal CT – lung windows – large amount of abnormal air in ascending aorta (AO). Aortic valve (AV) is delineated nicely demonstrated due to surrounding air.

**Figure 4.**
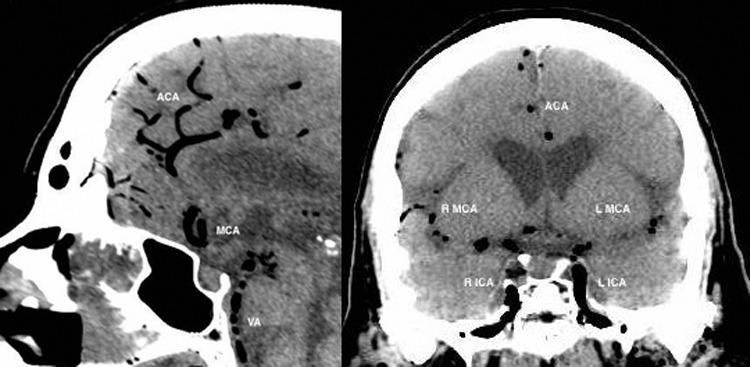
Sagittal and coronal CT head: abnormal intra-arterial gas seen within internal carotid arteries (ICA), vertebral artery (VA) and intra-cranial arteries. Air embolus is seen in bilateral peri-callosal anterior cerebral arteries (ACA) and bilateral middle cerebral arteries (MCA) (R = right; L = left).

**Figure 5.**
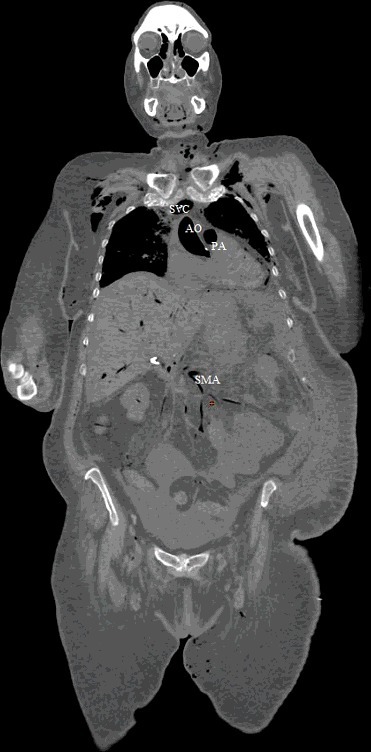
Coronal CT-soft tissue window: intravascular air in aorta (AO), pulmonary artery (PA), superior vena cava (SVC) and superior mesenteric artery (SMA). There is intravascular air in the liver and subcutaneous emphysema in the upper chest and neck soft tissues.

**Figure 6.**
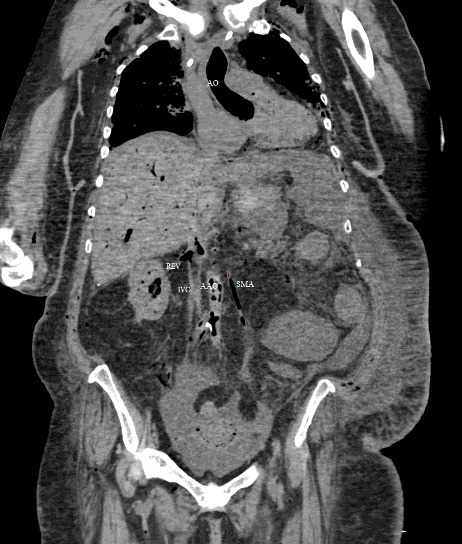
Coronal view (soft tissue window): intravascular air noted in the ascending aorta (AO), right renal vein (REV), inferior vena cava (IVC), abdominal aorta (AAO) and superior mesenteric artery (SMA).

**Figure 7.**
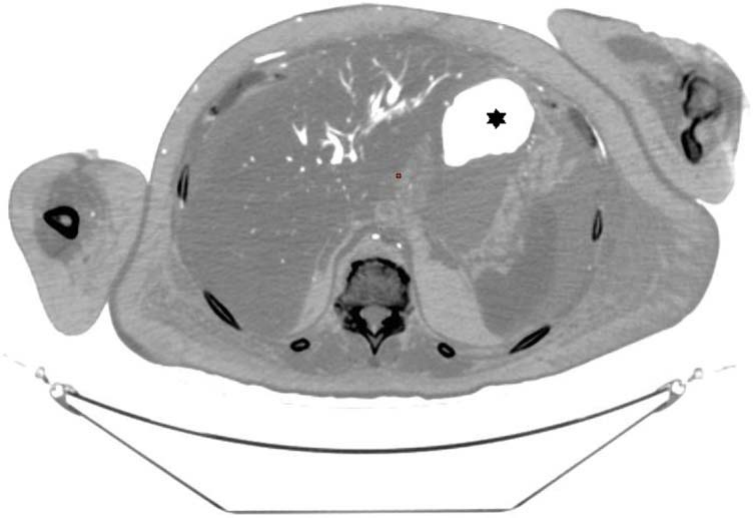
Axial CT (inverse grey scale): The hepatic parenchyma shows mostly intravascular air filling the intrahepatic vasculature and possibly minute dots of parenchymal air. The area marked with a star represents normal gastric air.

The PMCT showed large amounts of air in the right ventricle, right atrium, left ventricle, main pulmonary artery, aorta, and vena cava, intracranial, renal, hepatic, mesenteric, and proximal femoral vessels (Figures 2–6). There was only minimal intraparenchymal air in the liver noted (Figure 7). Air was also noted in the soft tissues of the chest (subcutaneous emphysema), due to multiple anterolateral rib fractures and fractured sternum as a result of aggressive resuscitation. Additionally, there was a moderate amount of ascites noted.

The significant autopsy findings were extensive adhesions and fibrosis in the upper abdominal region with extensive anatomic distortion, making the dissection of the vessels very difficult to impossible. The liver was firm and fibrotic, and there were moderate amounts of ascites in the peritoneal cavity. There were occlusive thrombosis of two hepatic veins, portal vein and a non-occlusive thrombus of the retrohepatic IVC. The review of other organ systems was unremarkable, except for mild left ventricular hypertrophy and cardiomegaly. There was no patent foramen ovale noted. Histologic examination of the liver was remarkable for non-occlusive, organizing thrombus of IVC and organizing and occlusive thrombosis of hepatic veins. The lungs showed extensive intravascular giant cell formation and granulomas containing polarizable material, hemosiderin and vegetable material surrounded by inflammatory cells. The cause of death was certified as air embolism complicating upper GI endoscopy. IVC and hepatic vein thrombosis, pancreatic carcinoma in remission (status post-Whipple procedure) and intra-abdominal fibrosis were listed as contributing factors. The manner of death was classified as natural.

## Discussion

Fatal and non-fatal air embolism caused by GI endoscopy and biliary system diagnostic or therapeutic procedures is a rare occurrence. Among GI endoscopic procedures, ERCP is most commonly associated with air embolism and the symptoms characteristically occur during the position changes from prone to supine [5]. Other reported endoscopic procedures associated with air embolism include sigmoidoscopy [10,11], colonoscopy [12,13], gastroduodenoscopy [14] and esophagogastroduodenoscopy [15–18]. Risk factors for developing air embolism that have been reported are prior surgeries of the bile duct system and transhepatic portosystemic shunt [6,19], blunt force trauma or penetrating hepatic injury [20]. Natural disease processes associated with an increased risk for developing air embolism have been reported to occur in certain conditions such as hepatic abscesses, inflammatory bowel disease, necrotizing enterocolitis, inflammation of the bile duct (cholangitis) or surrounding veins, mesenteric ischemia [21–23] and pneumatosis cystoides intestinalis with or without trauma among others [24–27].

The air introduced into the venous system may be limited to the portal vein and venous circulation or enter the arterial circulation and cause systemic air embolism, including the cerebral circulation. The amount of intravascular air in our case was very extensive, involving nearly the entire circulatory system.

Direct and inadvertent injury of either the venous or arterial circulation certainly can lead to air embolism during endoscopy or ERCP and is probably the most likely mechanism, especially if a sphincterotomy is performed during the procedure with resultant inadvertent vascular and mucosal injury. A vascular injury may occur by transecting delicate and abundant circulation of the small bowel at the papilla of Vater, or by direct instrumentation of the bile duct which can directly introduce air into the portal veins or IVC. Biliary-venous fistulas/shunts and portocaval collaterals are other possible mechanisms reported [6]. Many reported cases for developing systemic air embolism include intracardiac shunts, intrapulmonary right to left shunts, retrograde flow into cerebral veins through superior vena cava, or through pulmonary veins into the left atrium [23,28]. The most common intracardiac shunt is a patent foramen ovale [29,30]. Other shunts, such as atrial or ventricular septal defects, pulmonary shunts and arterio-venous shunts could potentially cause systemic air embolism.

A proper diagnosis of air embolism in forensic medicine has many important implications, not only from a civil litigation point of view, but also in criminal cases. In clinical medicine, air embolism can be confirmed with precordial ultrasound Doppler, transesophageal echocardiography and multi-detector computed tomography. In forensic medicine, identification of air embolism is not straightforward and is more complicated. Not only putrefactive gas production presents a challenge in differential diagnosis, but also certain other factors, such as sepsis and obesity can produce putrefactive gases faster.

It is crucial to anticipate the possibility of air embolism in suspected cases to prevent introduction of artifactual air. The presence of air in autopsy cases does not necessarily indicate air embolism and has to be differentiated from putrefactive changes. One of the earliest methods, described by Mercier [7] and still used, includes puncturing of the right ventricle after opening and filling the pericardial sac with water. The presence of air in the right side of the heart is interpreted as air embolism, if the same procedure, performed on the left ventricle, is negative for air. If air is present in both sides of the heart, then the results cannot be interpreted. This method, although easy to perform, cannot distinguish air embolism from post-mortem gas production and is therefore unreliable. Another method is the use of aspirometer first described by Dyrenfurth [8]. This tool enables aspiration and quantification of intracardiac air, which is then analysed by gas chromatography and has demonstrated as a valid method to differentiate putrefactive gas from air embolism [31]. The results of this method should be interpreted with caution as the amount of different gases in the heart blood can change rapidly. As pointed out by Groppi et al. in an experimental analysis with injection of O_2_/O_3_ in three bodies, the CO_2_ appeared in the heart blood immediately while O_2_ decreased rapidly and N_2_ remained relatively stable. These results could be misunderstood as putrefactive gas [9,32]. Carbon dioxide was used in our case as primary gas to dilate the intestine. The presence of high CO_2_ could possibly be misinterpreted as putrefaction. The combination of gases used, for example in scuba diving fatalities with barotrauma or endoscopy should be considered when interpreting the gas chromatography results.

Multi-detector PMCT is another method to identify air embolism and the distribution of gas in the body. Egger at al. investigated the incidence and distribution of post-mortem gas to identify factors to distinguish putrefactive gas from cardiac air embolism in 119 cases. Using a grading system of I–III, they interpreted the amount and location of gas in different locations of the body and concluded that the presence of large amounts of air in the right heart and no or minimal amount of hepatic parenchymal air must have originated from a process other than putrefaction [33]. Using the same grading system, the PMCT findings in our case was grade III for cardiac air and grade 0–I for hepatic parenchymal air (Figures 2, 5–7).

In the presented case, the patient had several risk factors as well as multiple possible mechanisms for developing air embolism: (1) the patient had a history of Whipple procedure and underwent radiation treatment. The combination of these two therapeutic modalities caused extensive fibrosis of the upper abdominal region and anatomic distortion, including altered papillary anatomy in the form of hepaticojejunostomy. Although fistulas or vascular shunts were grossly not identified due to dissection difficulty, these were the most likely aetiologies for systemic air embolism in this case. The patient's multiple episodes of occult upper GI bleeding are also suggestive of a vasculo-intestinal shunt/fistula. (2) The presence of mycotic and occlusive thrombosis of portal and hepatic veins could have caused collaterals or even arterio-venous fistulas. (3) The presence of cholangitis also weakens the mucosal barrier of the bile duct, allowing for the insufflated air to enter the blood circulation directly. (4) A hepaticojejunostomy is not a “true” sphincter like papilla of Vater and allows air to easily enter the bile ducts. (5) Extensive histologic pulmonary findings (granulomas, previous evidence of aspiration and fibrosis) could have caused intrapulmonary shunts allowing the venous air to enter the systemic circulation.

Identification of air embolism in forensic pathology is based on multiple factors and findings. The clinical presentation is the first important clue, which should prompt the pathologist to follow proper procedures. A chest X-ray should be performed to identify air in the right ventricle. If a CT scanner is available, it should be performed and is preferable to analogue chest X-ray. PMCT not only identifies air in the heart, but also cerebral air, distribution of air and intraparenchymal air to identify putrefactive changes. The interpretation of PMCT findings is not straightforward and should be done by a radiologist with experience in PMCT or a pathologist with experience in PMCT interpretation. The presence of air on PMCT or analogue X-ray is not diagnostic of vital air and should be confirmed by aspirometer and gas chromatography.

In our case, the diagnosis of air embolism was based on the result of echocardiography at the hospital (intracardiac air) and the amount and distribution of air on PMCT, using the grading scale described by Egger et al. An aspirometer would have been used, if the result of echocardiography was unknown.

In summary, this case demonstrates a rare complication of upper GI endoscopy. Therefore, pathologists should be aware of this rare complication and undertake the necessary steps to diagnose a possible air embolism.

## References

[1] Adams V, Guidi C Venous air embolism in homicidal blunt impact head trauma. Case reports. Am J Forensic Med Pathol. 2001;22:322–326. 11563750 10.1097/00000433-200109000-00026

[2] Halbertsma FJ, Mohns T, Bok LA, et al. Prevalence of systemic air-embolism after prolonged cardiopulmonary resuscitation in newborns: a pilot study. Resuscitation. 2015;93:96–101. 26092516 10.1016/j.resuscitation.2015.06.007

[3] Qazi AQ, Haider ZA, Najam Y Fatal systemic air embolism in a neonate after cardiopulmonary resuscitation. APSP J Case Rep. 2015;6:11. 25629000 PMC4288831

[4] Donepudi S, Chavalitdhamrong D, Pu L, et al. Air embolism complicating gastrointestinal endoscopy: a systematic review. World J Gastrointest Endosc. 2013;5:359–365. 23951390 10.4253/wjge.v5.i8.359 PMC3742700

[5] Mittnacht AJ, Sampson I, Bauer J, et al. Air embolism during sigmoidoscopy confirmed by transesophageal echocardiography. J Cardiothorac Vasc Anesth. 2006;20:387–389. 16750742 10.1053/j.jvca.2005.08.015

[6] Bisceglia M, Simeone A, Forlano R, et al. Fatal systemic venous air embolism during endoscopic retrograde cholangiopancreatography. Adv Anat Pathol. 2009;16: 255–262. 19546613 10.1097/PAP.0b013e3181aab793

[7] Mercier L Observation sur l'introduction de l'air dans les veines et sur la manière dont il produit la mort [Introduction of air into the veins and the manner in which it produces death]. Gaz Med. 1837;5:481–487.

[8] Dyrenfurth F Zur Technik der Feststellung des Todes an Luftembolie [The technique for determination of death due to air embolism] Int J Leg Med. 1924;3:145–146.

[9] Varlet V, Smith F, Giuliani N, et al. When gas analysis assists with postmortem imaging to diagnose causes of death. Forensic Sci Int. 2015;251:1–10. 25828953 10.1016/j.forsciint.2015.03.010

[10] Traiki TB, Baaj J, Al BA, et al. Fatal air embolism during sigmoidoscopy performed under spinal anesthesia. Anesth Essays Res. 2012;6:210–212. 25885620 10.4103/0259-1162.108336 PMC4173453

[11] Sopeña-Falco J, Poch-Vall N, Brullet E, et al. Fatal massive air embolism following diagnostic colonoscopy. Endoscopy. 2013;45(Suppl 02):E91. 23526536 10.1055/s-0032-1326254

[12] Chorost MI, Wu JT, Webb H, et al. Vertebral venous air embolism: an unusual complication following colonoscopy: report of a case. Dis Colon Rectum. 2003;46:1138–1140. 12907914 10.1007/s10350-004-7294-6

[13] Christl SU, Scheppach W, Peters U, et al. Cerebral air embolism after gastroduodenoscopy: complication of a duodenocaval fistula. Gastrointest Endosc. 1994;40: 376–378. 8056250 10.1016/s0016-5107(94)70080-x

[14] Maccarone G, Shakoor T, Ellis B Air embolism after percutaneous transhepatic biliary drainage and subsequent endoscopic retrograde cholangiopancreatography (ERCP). Endoscopy. 2011;43(Suppl 2):UCTN:E399. doi:10.1055/s-0030-1256943 22275020

[15] Niehaus K, Zamora E, Harkins M Fatal central nervous system air embolism during elective esophagogastroduodenoscopy. Endoscopy. 2013;45(Suppl 2):E205. doi:10.1055/s-0033-1344328 23852651

[16] McAree BJ, Gilliland R, Campbell DM, et al. Cerebral air embolism complicating esophagogastroduodenoscopy (EGD). Endoscopy. 2008;40:E191–E192. 18709611 10.1055/s-2007-995728

[17] López JC, Pérez X, Esteve F Cerebral air embolism during upper endoscopy. Endoscopy. 2010;42(Suppl 02):E41. 20073014 10.1055/s-0029-1215313

[18] Pandurangadu AV, Paul JAP, Barawi M, et al. A case report of cerebral air embolism after esophagogastroduodenoscopy: diagnosis and management in the emergency department. J Emerg Med. 2012;43:976–979. 21236613 10.1016/j.jemermed.2010.11.031

[19] Finsterer J, Stollberger C, Bastovansky A Cardiac and cerebral air embolism from endoscopic retrograde cholangio-pancreatography. Eur J Gastroenterol Hepatol. 2010;22:1157–1162. 20555267 10.1097/MEG.0b013e32833c5459

[20] Mohammedi I, Ber C, Peguet O, et al. Cardiac air embolism after endoscopic retrograde cholangiopancreatography in a patient with blunt hepatic trauma. J Trauma. 2002;53:1170–1172. 12478046 10.1097/00005373-200212000-00023

[21] Green BT, Tendler DA Cerebral air embolism during upper endoscopy: case report and review. Gastrointest Endosc. 2005;61:620–623. 15812425 10.1016/s0016-5107(04)02788-9

[22] Kennedy C, Larvin M, Linsell J Fatal hepatic air embolism following ERCP. Gastrointest Endosc. 1997;45: 187–188. 9041008 10.1016/s0016-5107(97)70246-3

[23] Lowdon JD, Tidmore TJ Fatal air embolism after gastrointestinal endoscopy. Anesthesiology. 1988;69:622–623. 3177925 10.1097/00000542-198810000-00032

[24] Hong JJ, Gadaleta D, Rossi P, et al. Portal vein gas, a changing clinical entity. Report of 7 patients and review of the literature. Arch Surg. 1997;132:1071–1075. 9336504 10.1001/archsurg.1997.01430340025003

[25] Kelly BJ, Meyers P, Choe KA, et al. Traumatic pneumatosis cystoides intestinalis with portal venous air embolism. J Trauma. 1997;42:112–114. 9003268 10.1097/00005373-199701000-00020

[26] Crausman RS, De Palo VA Pneumatosis cystoides intestinalis and portal venous air. Ann Emerg Med. 1998;31:286–287. 10.1016/s0196-0644(98)70332-4 9472198

[27] Gurland B, Dolgin SE, Shlasko E, et al. Pneumatosis intestinalis and portal vein gas after blunt abdominal trauma. J Pediatr Surg. 1998;33:1309–1311. 9722012 10.1016/s0022-3468(98)90176-2

[28] Desmond PV, MacMahon RA Fatal air embolism following endoscopy of a hepatic portoenterostomy. Endoscopy. 1990;22:236. 2242745 10.1055/s-2007-1012856

[29] Efthymiou M, Raftopoulos S, Antonio CJ, et al. Air embolism complicated by left hemiparesis after direct cholangioscopy with an intraductal balloon anchoring system. Gastrointest Endosc. 2012;75:221–223. 21470606 10.1016/j.gie.2011.01.038

[30] Park YH, Kim HJ, Kim JT, et al. Prolonged paradoxical air embolism during intraoperative intestinal endoscopy confirmed by transesophageal echocardiography -A case report-. Korean J Anesthesiol. 2010;58:560–564. 20589182 10.4097/kjae.2010.58.6.560 PMC2892591

[31] Bajanowski T, West A, Brinkmann B Proof of fatal air embolism. Int J Legal Med. 1998;111:208–211. 9646167 10.1007/s004140050153

[32] Groppi A, Mazza A, Papa P, et al. The analysis of the cadaveric gases in the medical-legal diagnosis of iatrogenic gas embolism. Int J Med Biol Environ. 1999;27:161–164.

[33] Egger C, Bize P, Vaucher P, et al. Distribution of artifactual gas on post-mortem multidetector computed tomography (MDCT). Int J Legal Med. 2012;126:3–12. 21207230 10.1007/s00414-010-0542-5

